# Passive myocardial mechanical properties: meaning, measurement, models

**DOI:** 10.1007/s12551-021-00838-1

**Published:** 2021-10-13

**Authors:** Ramona Emig, Callum M. Zgierski-Johnston, Viviane Timmermann, Andrew J. Taberner, Martyn P. Nash, Peter Kohl, Rémi Peyronnet

**Affiliations:** 1 Institute for Experimental Cardiovascular Medicine, University Heart Center Freiburg, Bad Krozingen, Freiburg, Germany; 2 Faculty of Medicine, University of Freiburg, Freiburg, Germany; 3 CIBSS Centre for Integrative Biological Signalling Studies, University of Freiburg, Freiburg, Germany; 4 Faculty of Biology, University of Freiburg, Freiburg, Germany; 5 Auckland Bioengineering Institute, The University of Auckland, Auckland, New Zealand; 6 Department of Engineering Science, The University of Auckland, Auckland, New Zealand; 7 Faculty of Engineering, University of Freiburg, Freiburg, Germany

**Keywords:** Young’s modulus, Nanoindentation, Hydrogels, Scar, Extracellular matrix, Fibrosis

## Abstract

Passive mechanical tissue properties are major determinants of myocardial contraction and relaxation and, thus, shape cardiac function. Tightly regulated, dynamically adapting throughout life, and affecting a host of cellular functions, passive tissue mechanics also contribute to cardiac dysfunction. Development of treatments and early identification of diseases requires better spatio-temporal characterisation of tissue mechanical properties and their underlying mechanisms. With this understanding, key regulators may be identified, providing pathways with potential to control and limit pathological development. Methodologies and models used to assess and mimic tissue mechanical properties are diverse, and available data are in part mutually contradictory. In this review, we define important concepts useful for characterising passive mechanical tissue properties, and compare a variety of in vitro and in vivo techniques that allow one to assess tissue mechanics. We give definitions of key terms, and summarise insight into determinants of myocardial stiffness *in situ*. We then provide an overview of common experimental models utilised to assess the role of environmental stiffness and composition, and its effects on cardiac cell and tissue function. Finally, promising future directions are outlined.

## Introduction: cardiac stiffness matters

The heart is a mechanically active organ with the amazing ability to contract in a rhythmic and well-coordinated manner, while its output continuously adjusts to circulatory demand on a beat-by-beat basis. The mechanics of active contractions have been the focus of numerous studies over many decades, which have driven progress in the understanding of cardiac pump function. However, pumping efficacy is not solely determined by contractile activity, but also by passive mechanical properties of the tissue, as these largely define the extent of chamber filling for example.

Recent development of techniques and models to better characterise and modulate passive mechanical properties of tissue and artificial substrates has led to major progress in the field of cardiac mechanobiology research. In this review, we clarify the terminology generally used to characterise passive mechanical properties, highlight why passive mechanics matters for cardiac function and present the main techniques used to assess tissue stiffness. We then provide an overview of passive mechanical properties of the myocardium in health and disease, and present key experimental and computational methods together with their applications. Finally, promising future directions for cardiac mechanobiology research and how they might aid the development of therapeutic approaches are outlined.

### Extracellular stiffness affects all cardiac cell types

The stiffness of the extracellular environment influences a host of cellular processes, including direction of cell migration, cell division and differentiation (Hinz et al. 2019, Fig. 1). Alteration of these cellular processes can lead to pronounced tissue remodelling, for example *via* altered fibroblast (FB) function, driving fibrosis (Herum et al. 2017). Passive tissue mechanics affect macrophage contributions to the healing process and subsequently scar formation or tissue composition (Atcha et al. 2021). Specific functions such as secretion (for FB, see Ceccato et al. 2020), extracellular matrix (ECM) modification and cell polarisation (for macrophages, see Dutta et al. 2020; Meli et al. 2020; Escolano et al. 2021; Atcha et al. 2021) are also affected by substrate stiffness.

**Fig. 1:**
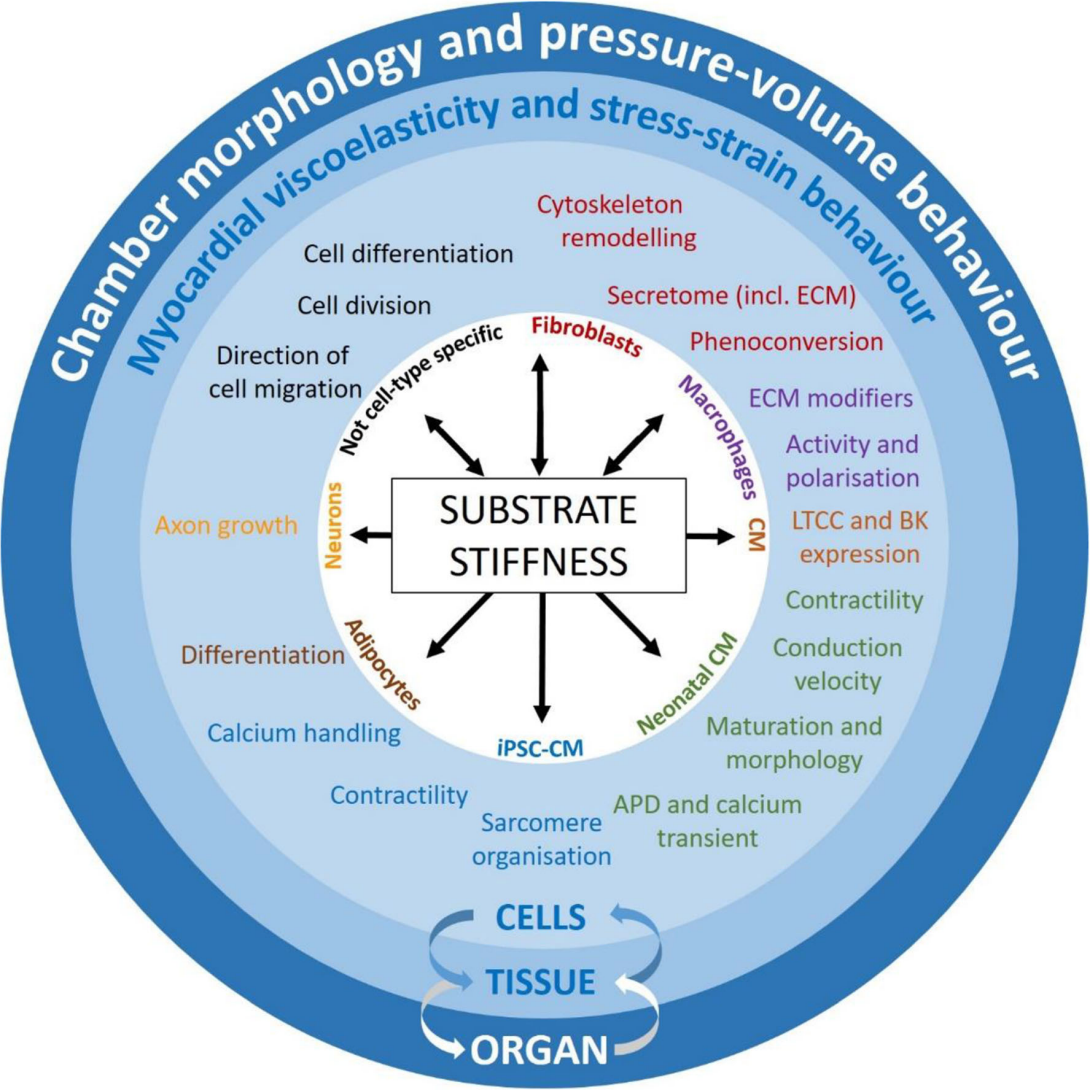
Main effects of substrate stiffness at cell, tissue and organ level. ‘Substrate’ includes both the native cell environment in tissue, which is largely determined by the extracellular matrix (ECM), and artificial matrices used as the foundation on which cells are kept in vitro. Other cardiac cell types, including smooth muscle cells, pericytes, endothelial cells, other immune cells, etc., are also likely to be affected by substrate stiffness. CM, cardiomyocytes; iPSC-CM, induced pluripotent stem cell-derived CM; FB, fibroblasts; LTCC, L-type calcium channel; BK, ‘big’ conductance potassium channel; APD, action potential duration

In cardiomyocytes (CM), ion channel expression changes in response to variations in matrix stiffness, including alterations in BK (the ‘big’ conductance potassium channel, Zhao et al. 2017) and L-type calcium channel expression. Cells in vitro, from induced pluripotent stem cell-derived CM to neonatal rat ventricular CM, also show adaptation in response to altered matrix stiffness, including modifications of structure, maturity, calcium handling and contractility (Ribeiro et al. 2015; Boothe et al. 2016; Corbin et al. 2019; Morrissette-McAlmon et al. 2020; Kit-Anan et al. 2021). Such adaptations are not unidirectional: cells whose activity is modified by substrate stiffness can alter the composition and/or organisation of the ECM, and hence change their own mechanical environment (Hinz et al. 2019). Such cellular adaptations to substrate stiffness are reflected at both tissue and organ levels, as discussed in the following section, all levels being interconnected (Fig. 1).

How cells sense biophysical stimuli in the first place, and how such stimuli are transduced into biochemical signals, has been a subject of research for decades (covered by several excellent reviews on cellular mechanotransduction in the past years: Ward and Iskratsch 2020; Angelini et al. 2020; Münch and Abdelilah-Seyfried^2021^; Stewart and Turner 2021). Here, we focus on experimental and computational methods to assess and to mimic passive mechanical properties, which is required for further progress in mechanotransduction research.

### Stiffness affects the myocardium as a whole

Passive myocardial stiffness affects the heart’s mechanical and electrical function (Jang et al. 2017; Nguyen-Truong and Wang 2018). Increased tissue stiffness (i) limits the speed and extent of diastolic relaxation and, thus, chamber filling and (ii) restricts CM contraction velocity and shortening amplitude. The orthotropic nature of cardiac stiffness is related to the complex myocardial tissue architecture, involving sheet-wise shearing motion of CM layers and re-orientation of sheetlets between more horizontal and more vertical orientations during contraction and relaxation, which is instrumental for wall thickening and thus efficient blood ejection (LeGrice et al. 1995, 2012; Hales et al. 2012).

Increased passive stiffness of the myocardium is thus associated with systolic and diastolic dysfunction (see review by Holmes et al. 2005). Systolic dysfunction can lead to chamber expansion, further increased wall stress and eventually heart failure with reduced ejection fraction (HFrEF). Changes in myocardial passive stiffness, by modifying how the tissue deforms upon stretch, can also alter active tissue responses to stretch, including changes in contractility (*e.g.* the Frank-Starling response; Frank 1895; Starling 1918), or electrophysiology (mechano-electric feedback; Lab 1996). These have recently been reviewed in detail elsewhere (Izu et al. 2020; Han et al. 2021; Quinn and Kohl 2021).

Diastolic dysfunction is associated with impaired tissue relaxation, potentially leading to heart failure with preserved ejection fraction (HFpEF). Conceptually similar observations on the interplay of tissue stiffness, systolic and diastolic function and chamber deformation were made in the atria (Allessie et al. 2002). Changes in tissue stiffness, thus, give rise to gross remodelling of cell properties, electrical coupling and ECM, and they affect the electrophysiological behaviour of atria and ventricles. The resulting electro-mechanical heterogeneities are highly arrhythmogenic, leading to electrical disorders in addition to the underlying mechanical dysfunction (de Jong et al. 2011). Although changes in tissue passive mechanical properties lead to alterations in both active and passive mechanics, only passive mechanics are reviewed here.

## Passive mechanical properties: key definitions

**Fig. 2:**
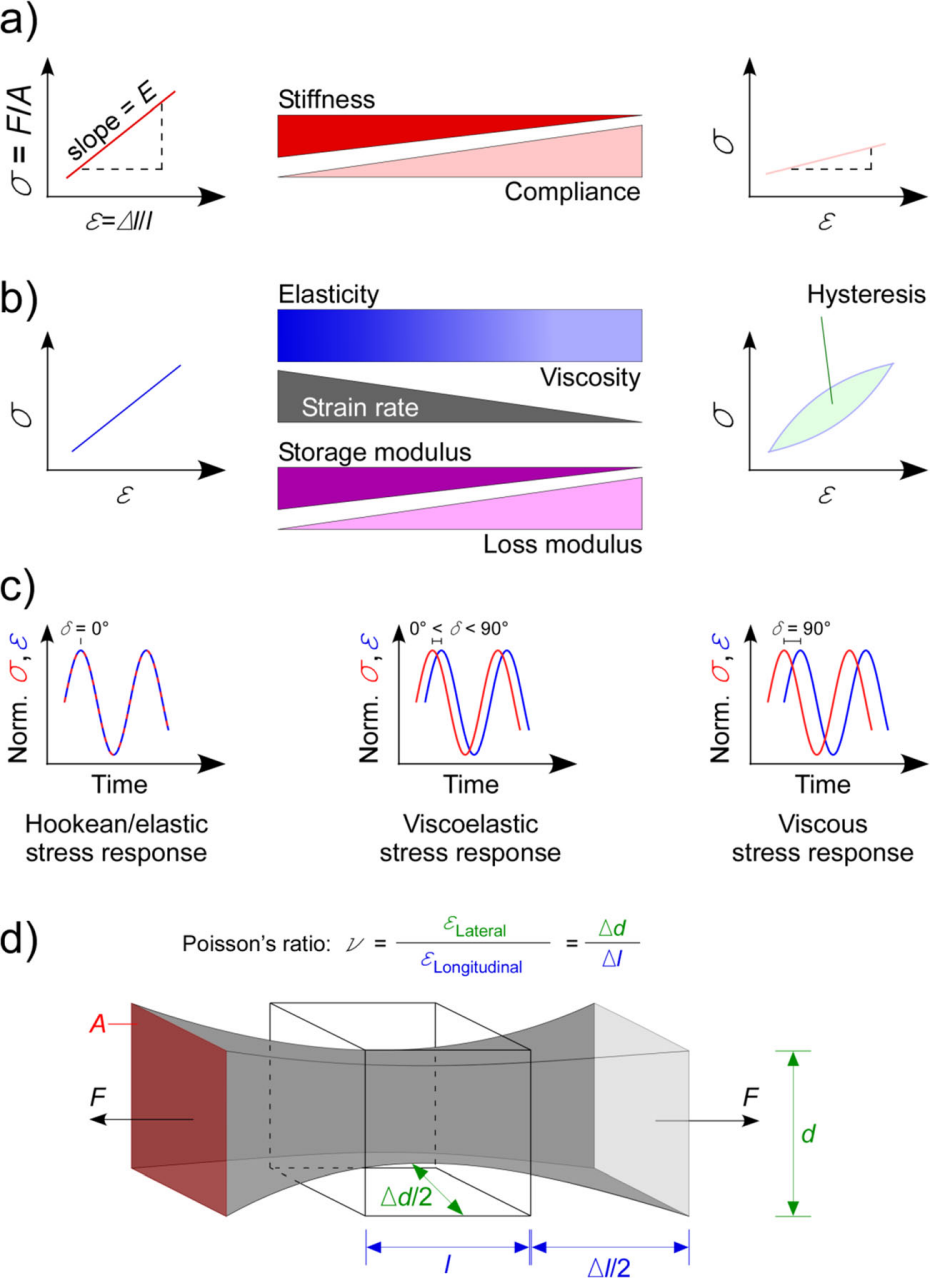
Key parameters describing passive mechanical properties. a Stiffness and compliance; b elasticity, viscoelasticity and viscosity; c dynamic mechanical analysis, σ and ε are normalised to ease reading the phase shift; d Poisson’s ratio. *σ*, stress; *F*, force; *A*, cross-sectional area; *ε*, strain; *l*, length in the direction of force application; Δ*l*, change in *l*; *E*, elastic or Young’s modulus; *δ*, phase shift; *d*, length in a direction normal to force application; Δ*d*, change in *d*

Misunderstandings often occur simply because terms used to describe tissue mechanical properties are not clearly defined and/or appropriately applied. It is common to examine only the ‘stiffness’ of a material, although there are many parameters that quantify mechanical properties (Table 1, Fig. 2). In this section, we will review definitions of important parameters that can be used to characterise passive mechanical properties, explain how they are interrelated and discuss the limitations of their applicability to biological materials.

**Table 1:** Key parameters describing passive mechanical properties

Parameter	Description	SI unit	Formula
Elastic modulus/stiffness	Resistance of a material to deformation	Pa	$E=\frac{stress}{strain}=\frac{\sigma }{\varepsilon }$
Compliance	Flexibility of a material	m/N (or 1/Pa)	$\mathrm{Compl}=\frac{strain}{stress}=\frac{\varepsilon }{\sigma }$
Viscosity	Resistance to flow	Pa*s	$\mu =\frac{F}{A}\ast \frac{dy}{du}$
Storage modulus	Ability to store elastic energy, a measure of elasticity	Pa	${E}^{\prime }=\frac{\sigma }{\varepsilon}\ast \cos \left(\delta \right)$
Loss modulus	Ability to dissipate energy, a measure of viscosity	Pa	${E}^{\prime \prime }=\frac{\sigma }{\varepsilon}\ast \sin \left(\delta \right)$
Damping	Relative degree of energy dissipation	Unitless	$\tan \left(\delta \right)=\frac{E^{\prime \prime }}{E^{\prime }}=\frac{G^{\prime \prime }}{G^{\prime }}$
Complex modulus	Resistance to deformation under vibratory conditions	Pa	*E*^∗^ = *E*^′^ + *E*^′′^
Poisson’s ratio	Negative ratio between lateral and longitudinal strain	Unitless	$\nu =-\left(\frac{\varDelta d}{d}\right)/\left(\frac{\varDelta l}{l}\right)$

*δ* phase shift, *du*/*dy* velocity gradient

To assess mechanical properties, one typically characterises the relationship between stress and strain in a material. *Stress* (*σ*) is the force (*F*) per area (*A*) within a material (Fig. 2a). Depending on the direction of external force application, tensile, compressive, shear, bending, torsional stress and fatigue can be differentiated. *Strain* (*ε*) quantifies the deformation as the change in length, Δ*l*, (or angle) as a fraction of the original sample dimension (Fig. 2a).

### Stiffness and compliance

The term most frequently used to describe the passive mechanical behaviour of biological materials is *stiffness*. Stiffness quantifies the extent to which an object resists deformation in response to an applied force. Compliance is the inverse of stiffness and quantifies the deformability of a material (Fig. 2a). Stiffness is a structural property of a given sample, and it depends on the material constituents of the sample, and their architectural organisation. The *elastic* or *Young’s modulus* (*E*) of a biological material is often referred to as a measure of ‘stiffness’, and characterises a material property that is independent of object geometry. Thus, *E* allows comparisons of stiffness properties between samples of differing shapes and sizes.

### Elasticity, viscosity and viscoelasticity

Elastic materials will fully restore their original size and shape after stress is removed. An elastic material can be regarded as a spring and its mechanical response may be described by Hooke’s law (Table 1; elastic modulus; Hooke 1678). The slope of the linear stress-strain relation is defined as the *elastic* or *Young’s modulus*(Fig. 2a, b). If a large stress is applied, the stress-strain relation may become *non-linear,* and the elastic modulus may change with strain. Moreover, the original size and shape may not be restored if the material is extended beyond the *yield strain*; this is referred to as *inelastic* or *plastic* deformation.

In a purely elastic material, the stress developed within the material depends solely on the *extent* of applied strain. However, many biological materials also exhibit resistance to the *rate* of length change; this is referred to as *viscosity*. Viscosity arises from friction-like interactions at the molecular level. The energy applied to viscous components in the material is not stored elastically, but is dissipated in the form of heat.

Most biological materials are *viscoelastic*: neither elasticity nor viscosity alone can sufficiently describe their behaviour (Fung 1993; Wang et al. 2016). For such a material, the stress-strain response may appear as a single curve when stress is measured after a series of discrete strains. When measured during an externally applied length change, a loop-like stress-strain pattern, known as hysteresis, is seen (Fig. 2b). While elastic components develop stress in phase with the applied strain, viscous components respond to the strain rate—the time derivative of strain. Assessing the properties of viscoelastic materials, especially for large strains, rapidly becomes complex with a wide range of possible models and parameters, as discussed in the “Limitations for biological samples” section.

Typically, experimental measurements of material properties are designed to simplify the analysis. Elastic properties are often measured by changing the sample strain and measuring the resulting steady-state force. Viscous properties can be quantified by measuring force and calculating stress at a variety of strain rates (*i.e*. speeds of stretch application). More sophisticated methods allow simultaneous measurements of elastic and viscous properties, using linear and non-linear system identification techniques, where strain is imposed using a signal comprising multiple different frequencies (Patra et al. 2020).

One such method is dynamic mechanical analysis (Fig. 2c). In these experiments, the material is subjected to frequency-rich perturbation of stress (or strain), while measuring the strain (or stress) response. The elastic component of material properties responds instantly to changes in stress or strain, whereas the viscous component induces a phase shift between the input and output signals. The ratio of stress to strain gives the overall resistance to deformation, known as the *dynamic* or *complex modulus* (E* or G*), depending on whether elongation/compression or shear is applied. The lag between stress and strain allows one to further break this down into the *storage* (*E*’ or *G*’) and the *loss moduli* (*E*” or *G*”). Both are frequency (*i.e.* strain rate)-dependent. The storage modulus quantifies the ability of a material to store energy elastically, while the loss modulus describes its ability to dissipate energy. Materials with a large storage modulus are generally regarded as elastic, whereas those with a large loss modulus are generally considered viscous (Fig. 2c, Patra et al. 2020).

Another method for assessing viscoelastic properties of a material involves *stress relaxation* experiments. In these experiments, strain is applied rapidly to the material and then held constant, while the stress is measured. In a viscoelastic material, stress is largest when the target strain is reached, and it will decline afterwards as the material relaxes, due to the rearrangement of viscous elements. Relaxation can be described by an exponential decay with the time constant of decay related to the viscous component,* i.e*. the more viscous a material, the slower it will relax and vice versa. In a comparable approach, a material may be subjected to a constant stress while monitoring strain. After the target stress is reached, strain can increase further at this constant stress, a process known as *creep*.

### Poisson’s ratio

*Poisson’s ratio* (*ν*) provides a measure of the strain that occurs in a material in directions normal to the applied force (Fig. 2d). Most materials have a Poisson’s ratio between 0 and 0.5, with 0.5 being characteristic of a perfectly incompressible isotropic elastic material. As cells and ECM components are commonly considered incompressible, their Poisson’s ratio is assumed to be 0.5 (Wells et al. 2015). Consequently, this is often applied to tissue. However, in anisotropic materials (*i.e*. myocardium, where mechanical properties are directionally dependent [see the “Anisotropy of (cardiac) cells and tissue” section]), Poisson’s ratio can exceed 0.5. It can also be negative, meaning that extension of the material in one direction leads to extension in other directions. Such property has not been described for cardiac tissue, but holds potential for cardiac tissue engineering, including the generation of cardiac patches (Kapnisi et al. 2018).

### Strength

*Strength* refers to the limit of stress that a material can withstand, and it is thus related to material stiffness. *Yield strength* is defined as the maximum stress that can be applied to a material before it undergoes plastic deformation, *i.e.* a deformation that is permanent, as the material will not relax to its initial configuration after the stress is removed. *Ultimate strength* is defined as the maximum stress that can be applied to a material before it fails, *i.e*. breaks. Measurements of stiffness of an elastic material, independent of the method used, are typically conducted well below the yield strength to ensure linearly elastic behaviour.

## Assessing mechanical properties from cell to tissue levels

**Fig. 3:**
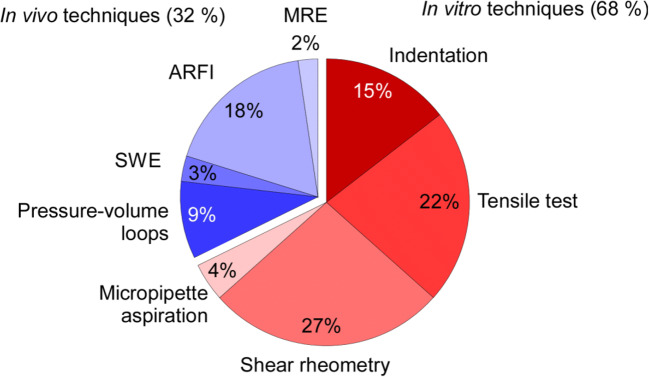
Relative use of the different techniques employed to assess mechanical properties of cardiac samples, from cells to whole organ. Numbers of articles (16,572 in total) found in Google Scholar using ‘Cardiac stiffness’ and the method name as search terms, excluding ‘kidney’, ‘liver’, ‘brain’, ‘aorta’, ‘vessel’, ‘tumor’, ‘plaque’, ‘clot’, ‘aortic’, ‘arterial’, ‘phantom’. SWE, shear wave elastography; ARFI, acoustic radiation force impulse; MRE, magnetic resonance elastography. Techniques used ex vivo are in red; techniques used in vivo are in blue

A variety of techniques are employed to assess the passive mechanical properties of biological samples (Guimarães et al. 2020). The relative frequency of use of these techniques in the context of cardiac mechanics in the literature is shown in Fig. 3.

### In vitro techniques

A range of measurement techniques can be applied to ex vivo cardiac tissues. These can reveal mechanical properties across a wide spatial scale, from isolated cells, through muscle strands, sections of myocardium, to the whole heart. Recent work highlights developments for the assessment of mechanical properties at the cell level (Narasimhan et al. 2020) even at high throughput (Romanov et al. 2021).

#### Indentation

Indentation experiments involve compressing a sample with known force (or displacement) and measuring the resulting displacement (or force). These measurements can be used to derive mechanical properties of the material. Indentation experiments are performed at various spatial scales, ranging from nanometres to millimetres. Nanoindentation, initially developed as a branch of atomic force microscopy, allows one to measure forces in the order of nanoNewtons with high spatial resolution (Qian and Zhao 2018). Indenters can employ minimised contact areas, down to a few square nanometres. This approach is convenient for measuring transverse mechanical properties of isolated cells and for assessing the local stiffness of various materials. Microindentators are physically larger and designed for the milliNewton and millimetre scales. These are useful for measuring mechanical properties of multicellular specimens and can be more easily combined with other experimental approaches, as their device foot-print is small compared to atomic force microscopes.

**Fig. 4:**
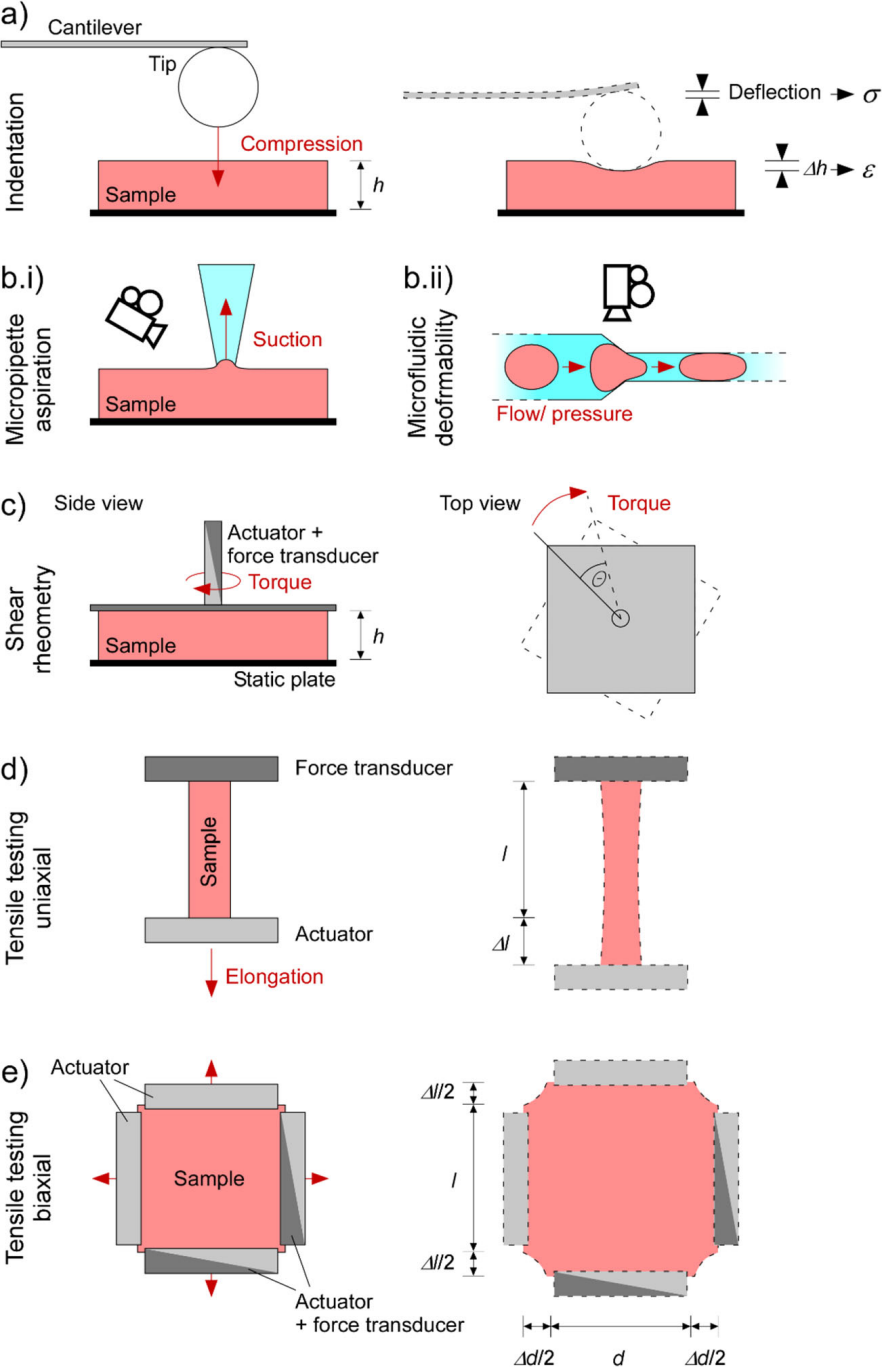
Illustration of in vitro methods, used to assess mechanical properties of cells and/or tissue. a (Nano-)indentation—the sample is compressed by the tip, leading to measurable deflection of a cantilever with known mechanical properties, which is used to assess indentation force. b Deformability assays—the sample is deformed by suction (micropipette aspiration, b.i) or pressure (microfluidics, b.ii), and sample (usually a cell) deformation is recorded optically. c Shear rheometry—rotational shear is applied to the sample by attaching it to two parallel plates and turning one of them, while measuring rotational force transmitted to the second plate. d and e Tensile testing—the sample is attached between two (uniaxial test, d), four (biaxial test, e) or more (not shown) sample holders and subjected to stretch along one or more axes, while recording the required force (reduction in sample thickness is not usually quantified). *h*, sample height; *θ*, angular displacement; *l*, sample length; Δ*l*, change in *l*; *d*, sample width; Δ*d*, change in *d*

Despite differences in detection methods and design of probes for contacting tissue, the rationale of indentation-based testing of mechanical properties applies across many systems (Ebenstein and Pruitt 2006). In most applications, a tip with well-defined geometry is attached to a pre-calibrated force transducer (*e.g.* a spring or a cantilever). The tip is used to contact and locally indent the sample, leading to a deflection of the cantilever (Fig. 4a), which allows one to document the force-indentation relationship of the material. The force-indentation relationship can be transformed into a stress-strain relationship by considering the geometry of the tip and using a model of how it contacts the tissue. The most common approach is the Hertzian model, which describes the contact between a sphere (the tip) and an infinite half space (the sample; Hertz 1881). For tips with other geometries, variations of the Hertzian model have been developed (*e.g*. the Sneddon model for cone-shaped tips). Major limitations of these models are their assumptions of homogeneity and infinite size of the sample, which are inherently inappropriate for biological samples. To overcome these limitations, more advanced models take into account a layered organisation of the sample and/or potential effects of underlying growth substrates (Gavara and Chadwick 2012; Managuli and Roy 2017; Rusaczonek et al. 2019).

Indentation testing can additionally reveal viscoelasticity of the sample. For example, maintaining strain at a specified state of indentation allows one to measure stress relaxation, while maintaining stress reveals substrate creep. Dynamic mechanical analysis is also possible with indentation to derive storage and loss moduli. However, given the anisotropy of cardiac samples, from single CM to the whole heart, the interrelation of ‘lateral’ indentation-based read-outs to properties relevant for ‘longitudinal’ force-length, or three-dimensional(3D)pressure-volume behaviour, is far from clear.

Indentation-based measurement can reveal spatially resolved information and potential heterogeneities within the sample. Consequently, especially in samples with a high degree of heterogeneity, large datasets involving surface scanning, together with model-based analyses, are required to draw conclusions on the mechanical properties of the whole sample. The information obtained by indentation is usually restricted to the surface of the sample and will be affected by heterogeneities in depth. Heterogeneities can manifest themselves from inherent sample topology, such as layered tissue arrangements, or arise as artefacts from sample preparation,* e.g. *cell death on the surface of a tissue slice.

#### Micropipette aspiration and microfluidic deformability

The assessment of mechanical properties using micropipette aspiration and microfluidic deformability assays is based on deformation of a cell when being forced through a small channel, either by suction (micropipette aspiration, Fig. 4b.i) or pressure (microfluidics, Fig. 4b.ii). While microfluidics-based methods are limited to single-cell analyses, micropipette aspiration has also been applied to valve leaflets (Zhao et al. 2011). Both methods are best suited for cells that natively occur in suspension, *e.g.* blood components such as erythrocytes and leukocytes, as their mechanical qualities may not be affected by the process of sample preparation. Adherent cells first need to be detached, which may alter their mechanics. Nonetheless, differences in mechanical properties between adherent cells after detachment have been detected (Tabatabaei et al. 2019), indicating that at least relative comparisons between mechanical attributes of cells isolated with the same experimental approach can be possible. Even for suspensions of cells, however, the mechanical features assessed by micropipette aspiration differ from those obtained by nanoindentation (Daza et al. 2019), probably because the mechanical properties of different structures are measured and strain is applied differently. These discrepancies are further underlined by the fact that the ability of cells to withstand suction *via* a pipette is mainly determined by the actin cortex just below the membrane (Pravincumar et al. 2012). Advances in micropipette aspiration, including biomechanical models for data analysis, have been reviewed earlier (González-Bermúdez et al. 2019). More recently, micropipette aspiration has also been used for dynamic mechanical analysis, and more advanced biomechanical models have enabled data analysis with sub-nanometre resolution (Berardi et al. 2021). Additionally, micropipette aspiration, when coupled to cytometry, allows one to sort cells based on their mechanical properties (Wang et al. 2013b) or, when coupled to impedance monitoring, to simultaneously record electrophysiological properties (Zhao et al. 2015; Wang et al. 2017), as reviewed earlier (Carey et al. 2019).

#### Shear rheometry

In shear rheometry, the mechanical properties of a material are tested by applying shear strain (*i.e*. movement in the plane of the sample contact surface) while measuring the resulting force. Sometimes, the sample is attached between two parallel plates, which are then moved with respect to each other (including rotational displacements). In other cases, a force/torque sensor can be used to apply shear forces/displacements to the surface of a tissue (Fig. 4c). As with indentation experiments, different protocols allow derivation of different mechanical properties. The slope of the shear stress vs shear strain relation is the shear modulus G* (analogous to the elastic modulus *E)*, while oscillatory perturbations allow the assessment of shear storage and shear loss moduli.

Samples often manifest strong anisotropy, which—in myocardium for example—reflects the orientation of CM and ECM. In such samples, the direction of shear with respect to the orientation of tissue components will affect the force that arises during shear, and thus the material properties measured in that direction. Furthermore, while indentation experiments reveal properties near the material surface, shear tests reflect the stress and strain developed across the whole preparation.

Local analyses require methods to measure spatially resolved strain. The measurement of surface strain (by optical tracking of intrinsic features or added markers) may be hindered by any attached plate or sensor. Measurements of internal strain usually require the integration of particles into the sample. While this may be an option for artificial materials, in particular if they are translucent, it is more difficult for cardiac tissue. One way to monitor deformation in native tissue is based on the implantation of high-density beads that can be tracked echocardiographically (Ashikaga et al. 2004). Other techniques allow strain tracking on and within cardiac tissue by imaging intrinsic tissue features, using modalities such as brightfield microscopy, confocal imaging, speckle tracking ultrasound, magnetic resonance imaging (MRI, Odening et al. 2013; Brado et al. 2017; Cheuk et al. 2021) or optical coherence tomography (Cheuk et al. 2017).

#### Tensile testing

Like shear rheometry, tensile testing reveals bulk mechanical properties of a material. In this method, a sample is stretched or compressed between an actuator and a force transducer. Length perturbations may be steps (revealing the elastic response and stress relaxation), or ramps, sinusoids or stochastic (revealing viscous responses). This approach can be applied to (quasi)two-dimensional samples like native tissue preparations (for example, tissue slices), engineered heart tissues, or artificial materials, and quasi-one-dimensional samples like trabeculae or individual cells.

Strain can be applied uniaxially or bi-/multiaxially (Fig. 4d, e). As the mechanical response of the tissue depends on the direction of strain with respect to CM and ECM orientation in the tissue, tensile testing often reveals anisotropy, such as along or across the locally prevailing CM orientation (see the “Limitations for biological samples” section). Biaxial tensile testing can reveal anisotropic mechanical properties that result from this structure. Moreover, assessment of local deformation by surface feature tracking allows one to analyse localised strain with high spatial resolution. When coupled with appropriate modelling techniques, this information can reveal the spatial heterogeneity and anisotropy of tissue stiffness across the sample.

### In vivo techniques with potential clinical application

In the clinical setting, measurement of passive heart mechanics is important for diagnosis and assessment of treatment effects, for example in diastolic dysfunction. However, in vivo measurements are hindered by the challenge of separating passive and active mechanics and by the lack of easy direct access to the myocardium. In practice, echocardiographic assessment of blood flow into the left ventricle is often used to diagnose dysfunction by assessing the speed and extent of diastolic filling. More advanced methods include image-based strain tracking of the myocardium itself, most often *via* ultrasound or MRI. These methods reveal local variations in diastolic and/or systolic strain, which can be used to explore regional differences in underlying passive mechanics, but these methods do not actually measure passive tissue stiffness (Brado et al. 2017).

Most methods for assessing passive myocardial stiffness in vivo quantify diastolic chamber stiffness, where a single parameter is given for a whole ventricle. A common method used to assess ‘chamber stiffness’ is by measuring pressure-volume loops and examining the end-diastolic pressure-volume relationship (EDPVR). Measuring pressure-volume loopes over a wide range of preloads to build up the EDPVR is difficult and time-consuming. However, recently, an approach towards single-beat estimation methods has been developed, to predict EDPVR from non-invasive single point measurements, making the concept much more clinically applicable (Chen et al. 2001; Klotz et al. 2006; Wang et al. 2018b). Diastolic chamber stiffness is altered by cardiac remodelling and can reflect diastolic dysfunction (Bastos et al. 2020). However, chamber stiffness has been found to depend not only on myocardial stiffness, but also ventricular geometry and functional changes in relaxation properties of the myocardium, which limits the applicability and usability of this measure (Romito et al. 2017; Wang et al. 2018b).

Computational modelling (see the “Data analysis” and “Computational modelling of passive mechanics” sections) can be used to provide a more accurate prediction of the underlying ventricular tissue stiffness. These methods use imaging techniques (MRI, ultrasound, etc.) in combination with 3D simulations of the contracting myocardium, to predict mechanical tissue properties (Wang et al. 2013c; Mojsejenko et al. 2015; Wang et al. 2018b; Palit et al. 2018; Rumindo et al. 2020). Computational models of MRI-based diastolic ventricular anatomy are constructed using finite element methods including either measured, predicted or generic prevailing CM directions to define tissue anisotropy. The finite element method is a numerical technique used to model the behaviour of a physical system, such as the deformation of a material (see the “Data analysis” section). This provides a powerful tool that can be used to represent cardiac mechanics while ensuring adherence to physical laws in a practical manner. Comprehensive reviews of the use of finite elements for analysing in vivo cardiac mechanics can be found elsewhere (Wang et al. 2015; Chabiniok et al. 2016).

Pressure conditions within the ventricle are defined either from assuming typical values for a particular patient cohort or in some cases from catheter measurements (Wang et al. 2018b). Computational models of mechanical contraction (see the “Data analysis” section) are then used with the defined shape and pressure conditions to predict how the myocardium will contract. Tissue displacement and wall thickening are compared to measurements, and diastolic material properties are modified in an iterative manner until the modelled displacements match those observed. These methods can indicate variations in myocardial stiffness across different patient cohorts. However, they typically provide only a single value and do not take into account local variations in tissue properties that occur in many disease states.

Direct measurements of local passive stiffness are important for separating out geometric or relaxation-induced effects, and for diagnosis or treatment monitoring where heterogeneity in stiffness may cause pumping deficiencies that could be masked by single parameter measures. Currently, there are three key methods for assessing local stiffness, acoustic radiation force impulse (ARFI), shear wave elastography (SWE) and magnetic resonance elastography (MRE). These methods estimate dynamic rather than quasi-static stiffness, resulting from ultrasound/MRI-based strain tracking approaches. ARFI, SWE and MRE are not currently in routine clinical use, but they hold great promise for providing locally defined stiffness values.

#### Acoustic radiation force impulse imaging

ARFI utilises an ultrasound transducer both for generation of acoustic pulses to apply force locally to the tissue and for tracking the resultant strains (for reviews on ARFI, see Nightingale 2011; Shiina et al. 2015). This method has the advantage that it can be performed with a standard ultrasound system, providing local stiffness assessment on a beat-by-beat basis. The most straightforward analysis method is to assess the strains resulting from ARFI, and so to obtain relative differences in tissue stiffness.

#### Shear wave elastography

A more quantitative approach is SWE, where the speed of shear waves resulting from a force impulse is measured. SWE can be achieved *via* application of an external force, or from shear waves that naturally result from closing of heart valves. The speed of shear waves is related to the shear modulus and, thus, the Young’s modulus. Calculation of the Young’s modulus requires the assumption that the heart is linearly elastic, which is reasonable for shear wave-based strains (≈0.1%), and that the bulk modulus is much higher than the shear modulus (typically true for biological tissues; Nowicki and Dobruch-Sobczak^2016^). The clinical utility of this approach has been examined for a number of organs and in chronic diseases; however, its application to cardiac mechanics remains limited (Bruno et al. 2016). Tissue penetration of ARFI is approximately 7 cm, due to dispersion and energy loss of the shear waves, limiting transthoracic imaging to the apex and ventral parts of the ventricles in larger mammals including humans (Bradway et al. 2007). Intracardiac echocardiography avoids this limitation, but makes procedures more invasive, and difficulties associated with correcting for catheter motion and imaging appropriate viewing planes limit applicability. This is exemplified by a study showing that, while altered stiffness associated with infarction of the left ventricular free wall could be identified, this was not possible in the septum (Hollender et al. 2013). Finally, ARFI requires extensive motion correction to ensure accurate measurements (Hsu et al. 2007), which is a key area for further development.

#### Magnetic resonance elastography

MRE operates on a similar principle as ARFI, but relies on an external actuator to apply vibrations to the chest. Resulting tissue displacements are measured using MRI (for review, see (Khan et al. 2018). MRE can be used qualitatively or quantitatively, with measurements based either on wave magnitude or speed, and subsequent conversion to shear modulus (in a manner similar to SWE). Validation of MRE in pig models showed clear differences in shear modulus between infarcted areas and remote myocardium. However, a number of the difficulties associated with ARFI also apply to MRE: signals diminish with increasing distance from the body surface, and tissue motion must be corrected for (Arani et al. 2017b). Moreover, MRE-based shear modulus values can be quite different from ex vivo uniaxial measurement of elastic modulus (although they follow the same trends), illustrating the difficulty of comparing data from different measurement methods (Mazumder et al. 2017; Arunachalam et al. 2018).

Thus, measurement of myocardial stiffness in vivo remains an important area of research and development. ARFI, SWE and MRE are promising approaches; however, their complexity still limits them to the preclinical setting, while issues such as limited penetration depth remain to be addressed. Common methods, used to assess mechanical properties in biology, are compared in Table 2.

**Table 2:** Common methods used to assess mechanical properties of biological materials from sub-cellular to whole organ level

Method	Strain	Parameters	Advantages	Limitations	Resolution	Types of sample
Indentation	Compression	*E*, compliance, *E*’, *E*”, stress relaxation, creep, adhesion	Applicable at various scales, spatially resolved force measurements	Only surface properties, high variability due to inhomogeneity of biological materials, affected by isotropy of cardiac tissue	μm to mm	ECM fibre, single cell, cell organelle, artificial material
					mm to cm	Whole heart, tissue, tissue section, engineered tissue, artificial material
Tensile test	Elongation/compression	*E*, compliance, *E*’, *E*”, stress relaxation, creep, strength	Applicable at various scales, spatially resolved measurements possible, integration of mechanical properties from all components	Affected by isotropy of cardiac tissue, sample geometry needs to well-defined, gluing or clamping can result in high stress regimes at attachment sites	nm to μm	Single cells, ECM fibre
					mm to cm	Tissue, tissue section, engineered tissue, artificial material
Shear rheometry	Shear (linear or rotational)	G*, *G*’, *G*”, stress relaxation, creep	Integration of mechanical properties from all components	Only bulk properties, requires very flat surfaces for attachment, affected by isotropy of cardiac tissue	mm to cm	Tissue, tissue section, engineered tissue, artificial material
Micropipette aspiration	Suction	*E*, compliance, *E*’, *E*”, stress relaxation, creep, strength	Can be combined with many other methods, *i.e*. electrophysiology	Attached cells need to be detached/isolated, affected by cell geometry	μm	Single cells, thin layers of tissue
Pressure-volume loops	Elongation	Stiffness, compliance	Easy comparison as only one value per heart/chamber is obtained	No spatially resolved information, differentiation between active and passive mechanics is difficult	Whole chamber	Whole body, whole heart
SWE	Shear	G* (derived from wave speed)	Non-invasive, commercial systems available	Significant post-processing required	mm to cm	Whole body, whole heart, tissue
ARFI	Displacement	Displacement in response to a known force ➔ *E*	Non-invasive, high resolution, commercially available systems, relatively easy interpretation	Qualitative, limited depth penetration	mm to cm	Whole body, whole heart, tissue, tissue section
MRE	Shear	G*	Non-invasive, high depth penetration	Values not comparable to other techniques	mm	Whole body, whole heart

*ECM*, extracellular matrix; *SWE*, shear wave elastography; *MRE,* magnetic resonance elastography; *ARFI*, acoustic radiation force impulse﻿

### Limitations for biological samples

Technical approaches to assess passive mechanical properties have been developed by physicists and material scientists, who are often working with more well-defined and less variable materials. These approaches, and the way the data are analysed, are based on a number of assumptions that are not necessarily applicable to biological samples. These include, but are not limited to, homogeneity, isotropy and passive nature of the material. Furthermore, ex vivo analyses of mechanical properties are hampered by the fact that biological tissue, once removed from its native mechanical environment, changes its properties. This effect is less dramatic when whole organ measurements are performed, but becomes quite pronounced when tissue sections, muscle bundles or single cells are studied.

#### Heterogeneity of biological materials

Biological samples are inherently heterogeneous at different scales. Tissues are composites of fibrous ECM with a variety of embedded cell types, each with different mechanical properties. The ECM itself is a polymer network, composed of a variety of fibrillary protein assemblies and crosslinkers. Even individual cells are not mechanically homogeneous as they contain a multitude of structures and organelles with distinct mechanical properties. Finally, not only the composition of the sample and the arrangement of all components, but also their interactions with one-other affect tissue mechanical properties. In practice, this is addressed by generating datasets using multiple methods for different samples across a range of scales, and interrelating results. More sophisticated and difficult approaches include finite element modelling, as described above (see the “In vivo techniques with potential clinical application” section).

#### Anisotropy of (cardiac) cells and tissue

In an isotropic material, mechanical properties are identical across all directions of strain. An anisotropic material has mechanical properties that vary depending on the direction of strain. An orthotropic material is a special case of anisotropy, where variations occur in three mutually orthogonal directions. The latter is generally the case for myocardium, which exhibits differing properties in the so-called fibre,^1^ sheetlet^2^ and sheet normal directions (see the “What contributes to myocardial stiffness” section).

While healthy myocardium can be considered orthotropic, a large degree of anisotropy—although not orthotropy—is observed also in scar tissue. Tensile testing can be employed to assess mechanical properties in the fibre and fibre-perpendicular directions. The importance of directionality is underlined by a study evaluating mechanical properties of rat myocardium in response to myocardial infarction (MI). Equibiaxial tensile testing revealed a continuous increase of the elastic modulus over 28 days following MI, when the tissue was stretched perpendicular to fibre direction, whereas no difference was found when stretching in parallel to fibre direction (Sirry et al. 2016).

#### Time-varying myocardial properties

Interpretation of measurements is further complicated by the fact that biological tissues are living materials, meaning that they change in composition and mechanics over time; they are therefore considered active matter. Biological materials may actively respond to mechanical stimuli that are applied during mechanical testing (*e.g.* stretch or compression). Responses to mechanical stimulation happen over different time scales, from alterations in CM myofilament calcium binding that may affect mechanical properties in milliseconds, over changes in calcium concentration or extension of actin fibres from existing monomers within seconds to minutes (Levin et al. 2020), to the expression, secretion and assembly of collagen fibres occurring over days and more (Schwarz 2015).

#### Comparability of data

Given the inherent anisotropy and complexity of cardiac tissue, and the significant effect of experimental settings and models used to analyse samples, caution needs to be taken when comparing results from different studies. The best option is to ensure well-defined constraints and repeatable approaches. Wherever this cannot be achieved, direct comparisons need to be handled with care.

### Data analysis

The interpretation of data from mechanical testing usually requires a compromise between simplicity and accuracy. The best approach depends on the kind of data collected and the questions that are to be addressed.

In some cases, a straightforward option is to assume that the material is homogenous and purely elastic, and to quantify its response by a single stiffness parameter, such as Young’s modulus. However, this approach is an oversimplification. A more rigorous approach is to use computational modelling techniques (see the “Computational modelling of passive mechanics” section for more detail) to construct a 3D finite element model of the sample, and to then iteratively change model material parameters until the predicted response of the model to loading matches that observed experimentally. This approach can characterise material properties with higher accuracy, but in most cases, it is impractical, particularly for applications where large sample sizes are required.

Analysis can separate the elastic and viscous components of the material properties. When considering elastic material properties, it is often presumed that materials are linear elastic; however, this is only the case over very small strains (<<1%). Most biological materials are hyperelastic, with non-linear stress-strain relationships that start linearly and then plateau. The material properties of hyperelastic materials are typically described by their strain energy density functions, which can be represented by a number of different material models. Neo-Hookean and Mooney-Rivlin models are the most well-known models for representing isotropic material properties (Mooney 1940; Rivlin 1948). However, there is a wide range of different material models that are more appropriate for representing the anisotropic properties of cardiac muscle. These come at the cost of increased complexity (for an overview of a number of models and their use for fitting soft tissue data, see Martins et al. 2006).

When viscous properties of the tissue are of interest, fitting data to a model taking into account both elastic and viscous material properties becomes necessary. To do so, a common simple approach is to represent the response by a series of springs (to account for elastic components) and dashpots (to account for viscous components) arranged in series and parallel with one another. The most basic model to describe viscoelastic behaviour is the Zener model (Zener 1948), which consists of two springs in series and a dashpot in parallel to one of the springs. Parametrisation of this model requires two elastic constants and one viscous constant, to describe the underlying material properties (assuming a linearly elastic, rather than a hyperelastic component).

Each data analysis method is associated with advantages and disadvantages, and these must be taken into consideration when choosing the methods to measure mechanical properties and to interpret the data. Further, when comparing data from different sources, not only the experimental settings, but also data analysis approaches need to be considered (see the “In vivo techniques with potential clinical application” and “Computational modelling of passive mechanics” sections).

## Myocardial stiffness in situ

### What contributes to myocardial stiffness?

#### Composition of cardiac tissue

The heart comprises a mix of different cell types, embedded within ECM. There are marked differences in cellular composition during development, between males and females (Squiers et al. 2020), between atria and ventricles of the same individual (Litviňuková et al. 2020) and between healthy and diseased tissue. Myocardial volume is generally dominated by CM (>60% of tissue volume, Mühlfeld et al. 2020). The remaining tissue volume comprises non-CM (such as FB, pericytes, neurons, endothelial cells, immune cells, adipocytes or smooth muscle cells), vascular (arterial, capillary, venous and lymphatic) lumens and ECM (the latter with a volume fraction of ≈2–7%, Díez et al. 2002; Borbély et al. 2005). Overall myocardial stiffness is a composite of cellular and ECM stiffnesses and their interplay. Another determinant of myocardial stiffness in vivo, which is often under-appreciated and hard to assess, is intra-vascular pressure. Increasing intra-vascular pressure has been shown to result in increased bulk stiffness of vascularised tissue (Livingston et al. 1994; Reeve et al. 2014).

#### Cellular stiffness

When considering cardiac cells only, CM play the most prominent role, even in diastole, as they occupy by far the largest part of the myocardial volume. Cellular stiffness of relaxed CM is largely dictated by the giant spring protein titin. Mutations leading to changes in titin’s mechanical properties are involved in different types of cardiomyopathy (Satoh et al. 1999; Gerull et al. 2002; Itoh-Satoh et al. 2002; Herman et al. 2012). Isotype switches between a stiffer and a more compliant isoform, and post-translational modifications, also change CM mechanical properties, contributing to cardiac diseases, as reviewed recently (Tharp et al. 2019). In addition to titin, CM contain several passive load-bearing structures such as microtubules, intermediate filaments and actin, which contribute to CM stiffness under resting conditions. All are subject to genetic and/orpost-translational modifications. Especially, detyrosination of microtubules is associated with changed mechanical properties and various cardiomyopathies (as reviewed by Ward and Iskratsch 1867).

The effect of non-CM on passive myocardial stiffness has gained little attention as (i) they occupy a comparatively small volume of the heart and (ii) their primary function, and thus their main contribution to mechanics, is thought to be through establishment and regulation of the ECM. The importance of their cellular mechanical properties may increase in diseased tissue where immune cells infiltrate the heart, and FB proliferate or phenoconvert into stiffer and more contractile myofibroblasts (MFB). This is difficult to disentangle from changes in ECM stiffness, as MFB are also the main drivers of collagen deposition and fibrosis. The role of FB stiffness for cardiac health has been underlined by a study showing that the elastic modulus of single ventricular FB in culture correlated better with left ventricular end-diastolic dimensions than the extent of fibrosis in tissue from recent-onset non-ischaemic cardiomyopathy patients (Glaubitz et al. 2014).

As non-CM are softer than CM, their stiffness *per se* is unlikely to be a major determinant of tissue stiffness. However, as essential modifiers of the ECM, they affect tissue stiffness indirectly. Further, cell stiffness (meaning cytoskeleton composition and organisation) contributes to the control of various cell functions such as differentiation, proliferation and secretion for non-CM, such as FB (Fig. 1). As cell stiffness affects MFB activity, adjusting ECM composition and rigidity, this may give rise to feedback leading to a further increase in tissue stiffness—a response that could be auto-regulatory by nature, if it helped to reduce the extent of excess strain in diseased cardiac muscle.

#### ECM stiffness

ECM comprises different proteins, including collagens, fibronectins, lamins and glycoproteins, which equip the ECM with distinct mechanical and biochemical properties. Often, tissue stiffness is inferred by analysing total collagen content in the ECM. However, this simplistic approach ignores differences between collagen subtypes, and the crosslinks that form between them and other ECM components and/or cells. Collagen I fibres are characterised by high tensile stiffness, while collagen III fibres are more compliant in ex vivo cardiac tissue (Collier et al. 2012); both can be stiffened in disease. One source of collagen fibre stiffening is fibronectin, which has been shown to be required for the generation of collagen fibres in vitro (Sottile and Hocking 2002) and for fibrosis development in mice in an in vivo model of MI/reperfusion injury (Valiente-Alandi et al. 2018). Collagen crosslinking is further mediated by glycoproteins which increase tissue stiffness due to advanced glycation end products (as seen in a model of volume overload, Herrmann et al. 2003). Importantly, ECM is a dynamic structure which undergoes constant remodelling. The role of different ECM elements, and how they are regulated, has been reviewed recently (Ward and Iskratsch 1867). Another review has focussed on how ECM adapts to different modalities of heart failure (HF, Frangogiannis 2019).

The effect of ECM on myocardial tissue stiffness was highlighted in studies that assessed the stiffness of whole and decellularised cardiac tissue in different species. Uni- and biaxial tensile testing revealed higher elastic moduli in decellularised compared to native cardiac tissue in rat (Ott et al. 2008; Witzenburg et al. 2012) and pig (Wang et al. 2013a). A more recent study shows that the mechanical properties of decellularised porcine tissue after introduction of induced pluripotent stem cell-derived CM are not different from native myocardium (Sewanan et al. 2019). This indicates that the differences between stiffnesses reported for native and decellularised tissue might be due to miscalculations, resulting from the porous structure of decellularised tissue. Similar comparisons on human tissue are lacking in the literature.

#### Tissue stiffness is a composite of cellular and extracellular stiffness

As detailed above, the main contributors to passive mechanical properties of the myocardium are CM, especially their titin filaments, and the ECM. Currently, a widely accepted concept is that titin dominates tissue stiffness at physiological strain levels, while the ECM network gets straightened/uncoiled rather easily. At larger strains, the influence from the ECM becomes increasingly more important, as collagen fibres are stretched to their full length (Granzier and Irving 1995; Granzier and Labeit 2004), preventing excessive strain of cardiac muscle. This can be imagined as two different springs, arranged in series, with collagen being more compliant at low strains.

The relative contribution of cellular and extracellular structures to overall tissue stiffness changes during disease. In healthy myocardium, the relative contribution of ECM to bulk tissue mechanics is lower than in tissue containing mature scar, months or decades after MI. During development of interstitial fibrosis, relative contributions of cellular and extracellular components to tissue stiffness change. This was demonstrated in a study on ventricular biopsies obtained from human patients, showing an increased contribution of ECM to myocardial stiffness in pressure- and volume-overloaded hearts, compared to controls (Chaturvedi et al. 2010). On the other hand, when CM become hypertrophic without the development of fibrosis, for example in response to volume overload, their relative contribution to bulk mechanics can increase.

In a mouse model of diastolic dysfunction, myocardial stiffness increase was carried more by changes in titin than ECM, *i.e*. the importance of CM stiffness increased in disease (Slater et al. 2017, reviewed by Franssen and González Miqueo (2016). Others reported increased collagen volume fraction and crosslinking in human patients (Kasner et al. 2011). None of these reports directly compared CM with ECM properties. The importance of their interplay for cardiac output in patients was shown in a study in which a combination of differences in passive CM tension and collagen volume fraction correlated best with left ventricular end-diastolic pressure (Borbély et al. 2005).

### Myocardial stiffness in health

**Fig. 5:**
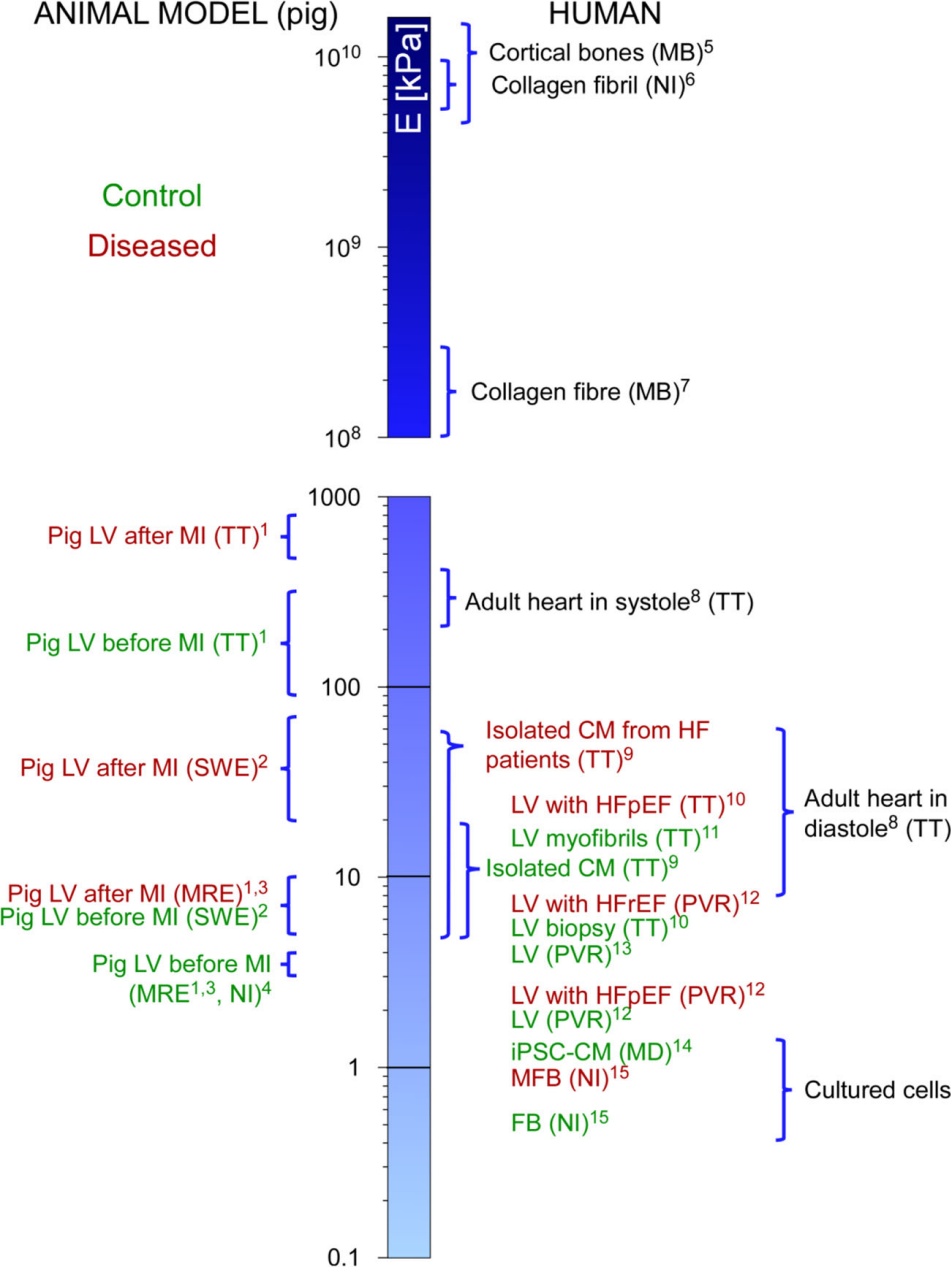
Cell and tissue stiffness in health and disease, with key reference points. Techniques used for the measurement are in brackets. *E*, Young’s modulus; LV, left ventricle; MB, microbending; TT, tensile test; NI, nanoindentation; MD, microfluidic deformability assay; MRE, magnetic resonance elastography; PVR, pressure-volume relationship; SWE, shear wave elastography; CM, cardiomyocyte; iPSC-CM, induced pluripotent stem cell-derived CM; FB, fibroblast; MFB, myo-FB; HF, heart failure; HFpEF, HF with preserved ejection fraction; HFrEF, HF with reduced ejection fraction. The only animal data considered here are from pigs, included because of their proximity to human cardiac structure. (1) Arunachalam et al. 2018, (2) Torres et al. 2018, (3) Mazumder et al. 2017, (4) Gluck et al. 2017, (5) Rho et al. 1993, (6) Wenger et al. 2007, (7) Dutov et al. 2016, (8) Huyer et al. 2015, (9) Caporizzo et al. 2020, (10) Zile et al. 2015, (11) Makarenko et al. 2004, (12) Wang et al. 2018b, (13) Arani et al. 2017a, (14) Pires et al. 2019, (15) Hoffmann et al. 2020

Comparing reported myocardial stiffness values (as shown in Fig. 5) requires caution. The wide diversity in Young’s moduli of individual components, contributing to tissue stiffness, is striking. Values in the 1 kPa range are characteristic of cultured cells, while elements of the ECM like collagen fibres or fibrils are characterised by stiffnesses of several GPa (Fig. 5; for further reference points, see Guimarães et al. 2020).

Although often regarded as a constant parameter, tissue stiffness is highly dynamic. The embryonic murine heart stiffens progressively, from < 1 kPa for the cardiac tube to 10 kPa in embryos aged E2 to E14 (Majkut et al. 2013, 2014; Chiou et al. 2016), as assessed by micropipette aspiration and computationally. In addition to the amplitude, these findings illustrate the impressive speed at which cardiac stiffness can change.

Concerning the adult heart, we focus here primarily on data obtained from human tissue; values from animal models can be found elsewhere (Liu and Wang 2020). In surveying studies of myocardial stiffness in human samples, a wide variability of results is evident, and often a stiffness range is reported rather than a single number. This variability stems from experimental conditions, techniques and the inhomogeneity of tissue samples to which investigators have access (high intra- and inter-patient variability, Ward and Iskratsch 1867).

The Young’s modulus of diastolic adult human myocardium is in the range of 8–15 kPa (Huyer et al. 2015; Arani et al. 2017a), as assessed with MRE and tensile testing. How this changes in disease is discussed next.

### Myocardial stiffness in disease

Fibrosis is the most common factor leading to changes in passive myocardial tissue stiffness, and it is part of the phenotype of most cardiac diseases (*e.g.* MI, ischaemic, dilated, diabetic, and hypertrophic cardiomyopathy, or atrial fibrillation). The few cardiac diseases from which fibrosis is generally thought to be absent are caused by genetic disorders, such as Brugada syndrome, long and short QT syndromes, as well as catecholaminergic polymorphic ventricular tachycardia. Whether interstitial fibrosis acts as a trigger, by-product or consequence of cardiac disease is a subject of research.

#### Tissue softening

Following acute injuries such as MI, catheter ablation or surgical lesions, cardiac cells and tissue undergo pronounced remodelling, forming a scar that prevents tissue rupture. While there are many studies on later disease stages, the early post-MI phase, when CM die, inflammation occurs and FB are activated and progressively replaced by MFB, has gained less attention from a mechanics perspective. The early post-MI phase involves degradation of ECM, mainly by metalloproteinases, which leads to a softening of myocardium, favouring inflammatory processes including macrophage infiltration. In a canine model, the stiffness of myocardium, assessed from *ex vivo*pressure-volume loops, decreased by 41% within 1 h after MI compared to sham-operated control hearts (Forrester et al. 1972).

Tissue softening has also been reported in chronic disease settings, such as for dilated cardiomyopathies (Nagueh et al. 2004). For a large part, these changes in stiffness are attributed to titin, rather than ECM. Indeed, titin truncation variants have been found in up to 27% of patients with dilated cardiomyopathy (Herman et al. 2012). Truncations are linked to reductions in elastic tension of up to 28%, based on tensile testing of isolated human cardiac myofibrils which are formed by repeating sarcomeres (Makarenko et al. 2004).

#### Tissue stiffening

Following the inflammation phase of MI, additional ECM is produced and tissue stiffness increases locally to levels similar to those prior to injury. As scars continue to mature, stiffness values exceeding 55 kPa (*i.e*. at least 5 times the stiffness of the healthy myocardium) have been observed in fully mature scars, months or years after injury (Holmes et al. 2005; Berry et al. 2006; Gluck et al. 2017; Farré et al. 2018). Interestingly, while this exceeds diastolic stiffness of healthy myocardium (in the range of 8-10 kPa), this value is below the stiffness values reached during systole (> 100 kPa, Huyer et al. 2015). It is evident therefore that the cardiac scars, formed as part of the hearts emergency repair and protection programme, may show ‘paradoxical segment lengthening’ (*i.e.* positive strain during mechanical systole), even after full maturation (even if that may be difficult to detect using clinical strain tracking), which would have negative implications for cardiac pump function.

Chronic injuries can be due to mechanical overload, as occurs in dilated and ischaemic cardiomyopathies, valve defects or hypertension, and this can lead to diffuse fibrosis. This fibrosis pattern has less pronounced effects on stiffness than that observed in mature scars, but it can still severely alter tissue mechanics, electrics and function, changing the mechanical microenvironment of individual cells. This can result in dysfunction of cardiac cells (see the “Extracellular stiffness affects all cardiac cell types” section). If diffuse fibrosis affects overall organ function, a vicious cycle towards maladaptive remodelling of cardiac tissue can be initiated (both in ventricles and atria). Such deterioration of tissue function contributes to disease progression (as in HF and atrial fibrillation, for example). The case of HF, combined with hypertension, is peculiar and was the focus of detailed studies.

Hypertension in the absence of HFpEF does not alter passive myocardial stiffness of the human left ventricle (Zile and Baicu 2015). In contrast, patients with hypertension *and* HFpEF show a significant increase in passive myocardial stiffness, from *E* between 9 and 10 kPa for controls to 25 kPa for patients with hypertension and HFpEF. Both ECM and titin-dependent stiffness are increased, with collagen appearing to be the main load-bearing structure (Zile and Baicu 2015). These data suggest that the development of HFpEF is dependent on changes in both collagen and titin homeostasis. Using advanced biomechanical models to derive LV tissue stiffness from catheter measurements, another study suggests that diastolic tissue stiffness is significantly higher in HFrEF patients compared to HFpEF and control patients (Wang et al. 2018b).

With elevated end-diastolic pressure, higher ventricular tissue stiffness has been observed in pressure-overloaded, but not in volume-overloaded hearts (Chaturvedi et al. 2010). Both ECM and CM are thought to contribute to this. The study was based on biopsies obtained from patients with congenital heart disease as models for hypertrophy caused by pressure overload or ventricular dilatation (Chaturvedi et al. 2010).

We detail the above reports, as data on human tissue are scarce, and cardiac samples will always remain difficult to access for direct investigations. For this reason, research combining experimental and computational models remains to be particularly useful.

## Experimental and computational models to assess the role of environmental stiffness on cardiac cells

*In vitro* models represent a trade-off between resembling physiological conditions as closely as possible and allowing flexibility (in terms of control over relevant input parameters and ease of observation of read-out parameters) to study mechanisms and (potentially causal) chains of events in depth. This following section will focus mainly on approaches that allow one to actively modify mechanical properties of the in vitro environment (the growth matrix), and briefly touches on approaches that are designed to resemble as closely as possible the native structural and mechanical properties of cardiac tissue.

### Hydrogels to study the effects of matrix stiffness on cardiac cell function

When looking for a hydrogel model as a growth substrate for cardiac cells, one has the choice between hydrogels based on artificial polymers, including polyacrylamide, polyethylene glycol, polydimethylsiloxane, *etc*., and naturally occurring ECM proteins, including collagen, hyaluronic acid, *etc*. While artificial materials permit insertion of additional components, giving the hydrogel special properties (*e.g*. light tunability), they require the addition of adhesion molecules to allow cell attachment. Also, they tend to lack the highly ordered structure of myocardial tissue. In contrast, hydrogels based on natural ECM components are well-suited for cell attachment without further modification and may incorporate structural features. However, more complex interventions to dynamically, reversibly, precisely and reproducibly control mechanical properties of the growth substrate are difficult. Several systems have been developed that aim to combine the two approaches.

#### Hydrogels with varying elastic moduli

Polyacrylamide gels are frequently used as in vitro growth substrates to mimic different mechanical environments. Their elastic modulus can be modified by varying the ratio between acrylamide and its crosslinker (bis-acrylamide). They can be produced to cover a large range of stiffnesses and may include a number of different coatings/adhesion molecules, making their use attractive for many applications (Tse and Engler 2010). Being purely elastic, however, they do not represent the mechanical properties of biological materials, which are usually viscoelastic (Chaudhuri et al. 2015, 2020). By incomplete crosslinking, viscoelastic polyacrylamide gels can be prepared that vary their viscoelasticity and stiffness based on the degree of crosslinking (Cameron et al. 2011; Charrier et al. 2018). Recent developments allow one to control viscoelasticity or stress relaxation, as reviewed elsewhere (Chaudhuri et al. 2020). Such matrices have been successfully applied to cell culture experiments, showing that passive mechanical properties of the growth substrate influence cell functions (see the “Extracellular stiffness affects all cardiac cell types” section), and that both the elastic and the viscous component are essential mechanical inputs sensed by cells.

#### Hydrogels with dynamically tunable stiffness

There is considerable interest in tuning the mechanical properties of growth substrates, in the presence of cells, with good spatio-temporal control to assess cellular adaptations to their mechanical environment in real time. Many hydrogels are referred to as ‘tunable’ in the literature, simply because different stiffnesses can be obtained during curing. In the following, the term ‘tunable’ will be reserved for hydrogels whose mechanical properties can be changed in a contact-free manner, *after* the matrix has been cured, and in the presence of cells. These changes should be reversible, repeatable, stable without continuous stimulus and achievable over a large dynamic range.

Several strategies to dynamically tune the mechanical properties of cell culture substrates have been developed in the past decade. Some approaches, including thermo-sensitive(Teichmann et al. 2013; Uto et al. 2014), chemically tunable (Liu et al. 2018) and pH-responsive hydrogels (Osorio et al. 2007) are hampered by potential side effects from the ‘tuning signal’ itself on the cells. Other systems, based on stimuli which are not directly sensed by cells, like light or magnetic fields, are more suitable for biological experiments.

Magnetically tunable matrices result from equipping a basic hydrogel, with magnetic particles (Corbin et al. 2019). The elastic modulus of the matrix depends on the strength of an applied magnetic field and can be tuned between 6 kPa (no magnet) and 20 kPa (magnet as close as possible to the gel). This system has been applied to show differences in Yes-associated protein (a mechanically triggered transcription factor) localisation in CM and cardiac FB, MFB phenoconversion, iPSC-CM sarcomere organisation and expression levels of mechano-sensitive proteins (Corbin et al. 2019).

In light-tunable hydrogels, the crosslinking state of a polymer (and thus the stiffness of the hydrogel) can be altered by illumination. These gels comprise two groups: (i) the polymerisation state of the polymer is affected directly by a photochemical reaction, or (ii)photo-sensitive proteins, which undergo multimerisation upon illumination, are used.

Photo-initiated crosslinking often is not reversible (*e.g*. Nguyen et al. 2012; Yuan et al. 2018; Wang et al. 2018a) with the exception of an azobenzene-polyethylene-glycol hydrogel, which can undergo multiple gel-sol transitions (Accardo and Kalow 2018). Advances in the development of photo-crosslinkable hydrogels with a focus on biomedical applications have been reviewed recently (Choi et al. 2019).

Conformational changes leading to multimerisation of photo-sensitive proteins are usually reversible. Various photoreceptors have been used in combination with different polymers and adhesion molecules to obtain cell culture matrices (Zhang et al. 2013; Liu et al. 2018; Hörner et al. 2019). The essential limitation of currently available photoreceptor-based hydrogels for use in cardiac mechanobiology research is their comparatively low stiffness (<1 kPa to 10 kPa) and the limited dynamic range (≈two-fold). These are suitable for studies on brain and lung cells, or to study effects of regional tissue softening, such as early after MI. Cells from many tissues have been shown to be sensitive to currently available stiffness ranges, even though they are sometimes far from their native environment. Besides being non-invasive, the main advantage of using light as a stimulus for matrix modification is the possibility to generate spatially heterogeneous matrices with high precision.

Changing the elastic modulus of a viscoelastic matrix often also changes the loss modulus, making it impossible to disentangle viscous from elastic effects. Nonetheless, a number of studies based on static hydrogels with equal elastic, but different loss moduli, highlight the importance of viscoelasticity for tuning mechano-adaptation in FB (Chaudhuri et al. 2015; Lou et al. 2018; Ma et al. 2020). To date, there is no system that allows one to dynamically tune one or the other property independently in the presence of cells.

### Nanofibrous materials to mimic structural characteristics of the ECM

Cardiac tissue is highly anisotropic due to the organisation of CM and the ECM. This cannot be mimicked by hydrogels, in which a polymer and a crosslinker are mixed homogeneously. Thus, various biofabrication techniques for the generation of nano- or microfibrous structures have been developed. These include electro- or pull spinning (MacQueen et al. 2018), 3D bioprinting (Lee et al. 2019), micro-moulding or photo-patterning, as reviewed recently elsewhere (Elkhoury et al. 2021). Some of these can be combined with previously discussed artificial or natural hydrogel materials. While micro-moulding and photo-patterning provide additional mechanical cues by confining the growth space of the cells, 3D printing and electrospinning are used to capture heterogeneities in tissue structure in a more advanced way.

In 3D printing, the initial matrix is usually homogeneous, but by combining multiple inks with distinct mechanical properties, complex mechanical heterogeneities can be obtained. Other approaches feed the real 3D structure of a heart into the printing programme. In doing so, a hydrogel scaffold in the shape of a full-size human heart has been printed (Mirdamadi et al. 2020). More work is required, though, to establish a way to reliably populate such large preparations with the various cardiac cells types (Guyette et al. 2016; Mirdamadi et al. 2020), and to establish a system of perfused 'vessels' to mimic the coronary vasculature. Spinning of nano- or microfibres represents an intriguing technique to resemble the fibrillary architecture of the ECM (Morrissette-McAlmon et al. 2020). All these techniques represent promising candidates for the generation of cardiac patches for future therapeutic approaches (Nguyen-Truong et al. 2020; Beck et al. 2021).

Instead of artificially building a fibrous matrix, other approaches make use of the cells’ own ability to form ECM, aiming to capture their native biochemical and mechanical properties. For these approaches, cells (usually multiple different cell types) are seeded in a 3D gel-like mixture of ECM proteins which they then assemble into fibrous ECM themselves. During the development of such engineered heart tissue in the past years, the importance of passive mechanical properties for cell functions has become increasingly clear. When the stiffness of a preparation is too high or too low, cell alignment is impaired, leading to decreased performance (Eschenhagen et al. 1997; Boudou et al. 2012). Even closer to physiological ECM structures are experimental models based on decellularisation of cardiac tissue, which leaves behind only the ECM (Ott et al. 2008). It is under debate, also, whether the decellularisation process changes the mechanical properties of the ECM (Witzenburg et al. 2012).

Although the above tools can closely mimic physiological structure and mechanics of the ECM, their main limitation for cardiac mechanobiology research is that the mechanical properties of these materials, once established, cannot be modified. To date, there is no system available that combines nanofibre spinning or 3D bioprinting with magnetically or light-tunable properties.

### Computational modelling of passive mechanics

The first mathematical models of processes describing muscle contraction were proposed in 1938 by A.V. Hill (Hill 1938). Hill’s fundamental model for muscle mechanics relates force development with the velocity of contraction using a hyperbolic function, while also incorporating energy consumption and muscle work. The phenomenologically derived Hill equation has since been used to describe muscle-specific characteristics of the force-velocity curve. Nearly 20 years after Hill’s experimental and mathematical model, A.F. Huxley published the first nano-structural, biophysically detailed crossbridge kinetics model of tension generation (Huxley 1957). With the technical progress of experimental metrology and increasing computational power during the subsequent 60 years, cardiac mechanics models have gradually been refined. Computational research now spans from nano- to macro-scales, combining knowledge from experimentation at multiple levels, such as models of length-dependent activation (Guccione et al. 1991; Hunter et al. 1998; Campbell 2011), thin filament (Julian 1969; Land and Niederer 2015)and/or thick filament regulation (Huxley 1957; Mijailovich et al. 2017) and ventricular mechanics (Arts et al. 1979), which has been recently reviewed (Niederer et al. 2019).

Mathematical functions used to simulate experimental results aim to capture the underlying mechanisms of biological processes. Before the advent of high-performance computing, a process was evaluated for the presence of exponential behaviour by testing whether data points, drawn semi-logarithmically, could be interpolated by a straight line. This method is a simple way to mathematically approximate a function without using a computer. Nowadays, with increasing amounts of data, computer models have become more sophisticated and represent the detailed structure and biophysics of cardiac tissue. As such models are generally too complex to be analysed 'by hand', computer simulations are necessary to interpret the available experimental data.

Research focussing on digital modelling of passive cardiac mechanics and contraction tries not only to describe the underlying physical characteristics of the biological system, but also to explain experimental observations. System-describing mathematical equations can be used to integrate data from different sources and interpolate data across scales and/or species to investigate the effects of sub-cellular biological mechanisms on whole-heart function (see also the “In vivo techniques with potential clinical application” section). This direction of research has a long history, including work by the physiologist Otto Frank who had captured cardiac pressure-volume relations in a mathematical model in 1895 (known as the fundamental principle of cardiac mechanics, this was later extended and called the ‘Frank-Starling law’, Frank 1895). To-date computational models can describe isolated cell mechanics with impressive detail. For example, the focal adhesion protein vinculin has been related to impaired cellular mechanics, which may lead to cardiac dilation and hypertrophy in humans (Zemljic-Harpf et al. 2009), a complex and dynamic process involving an increase in cavity volume and tissue mass of the heart. The effects of vinculin on the cytoskeleton are well understood, while growth models are complex and computationally expensive to analyse, and thus efforts to investigate cardiac hypertrophy have required several simplifying assumptions (Yoshida and Holmes 2021). A great challenge for whole-heart simulations is to reproduce the complex interrelation of cellular and ECM mechanics. This challenge remains insufficiently addressed.

Experimental approaches to tease apart mechanical effects at the inter-and/or intracellular levels and the ECM are limited. Simulations incorporate various types of information, from mechanical changes to influences of/on electrophysiology, energetics and even hormones or drugs, which can be advantageous but often also has drawbacks since simulations can only be as good as the experimental data with which they are parameterised. Thus, integrating experimental data from different sources potentiates biological variability and errors. Furthermore, results obtained in one species, such as mouse, have to be extrapolated to other species with great care (including ‘translation’ to humans—a precondition for utility in the context of clinical questions—as information based on human data is limited). Combining data from various species may lead to species non-specificity and uncertainties of the predictions.

At the whole organ level, computational models are further limited by computational power and mathematical methodology. But more importantly, current models do not intrinsically replicate whole-heart motion without kinematic constraints that, for example, limit the displacement of the epicardium. Hence, investigations of cardiac mechanical function at different scales are still needed. While computational models can be used for testing different hypotheses and examining the underlying mechanisms, experimental limitations are passed on to the computational examination and may be exacerbated by modelling and/or measurement errors. Furthermore, computational models can only approximate observed experimental observations and may require the introduction of apparent kinetics or dynamics to the model, to reproduce experimental observations (as done, for example, in the active mechanics model of Rice et al. 2008). Finally, simulations use reductive approaches to lessen dimensionality, spatiality and complexity due to limitations of computational capacity, which impairs model precision and introduces additional mathematical errors to the models.

## Applications and future directions

Our understanding of the passive mechanical properties of myocardial tissue, and their importance for cardiac function, is continuously expanding in line with the development and application of new strategies for tissue testing and characterisation. A wide variety of tools have been developed to probe passive mechanical properties of the heart in vitro and in vivo. Each tool and technique offers distinct advantages and disadvantages, with in vitro methods providing high experimental flexibility and precision while being far from native tissue settings, whereas in vivo methods combine potential clinical relevance with reduced control and. While a number of studies have assessed elastic moduli of cardiac tissue using more than one method, they have tended to compare different in vitro (or different in vivo) methods to one another, rather than comparing in vivo with in vitro approaches on matched or identical samples*.* The latter comparison will be required to improve our understanding of cardiac mechanics in clinical scenarios, and the interrelation of parameters assessed in and ex vivo.

As the cardiac mechanics research community develops, involving scientists from different fields (material scientists, engineers, biologists, clinicians), it is becoming ever more important to be consistent with the use of terminology, to avoid misunderstandings. With the emergence of new techniques, which allow more precise and comprehensive investigations, our analyses should go beyond simple reporting of the Young’s modulus, and consider the non-linearity and viscoelasticity of biological materials. In particular, disentangling stiffness and viscosity will be instrumental to better characterise cell and tissue-level responses to the mechanical cues affecting cardiac structure and function.

An important target is to develop efficient methods and treatments to slow, stop or reverse fibrosis, ideally in an organ- or even region-targeted manner, to remove undesirable side effects and heterogeneity. To do so, it is essential to assess the spatial and temporal heterogeneities in stiffness that occur in various disease settings. This requires an increased uptake of methods, such as ARFI, SWE and MRE, to obtain localised measurements of tissue stiffness, as well as computational models to fill the gap between structural and functional data and to provide quantitative insight from in vitro data on intra- and intercellular mechanics—ultimately projecting to in vivo organ level information.

A mechanistic understanding requires the teasing apart of various factors that define and alter tissue stiffness, which can then be used to inform conceptual models and develop treatment approaches. To this end, many in vitro models mimicking different aspects of tissue mechanics have been developed. These are, however, usually limited either by lacking structural features (*e.g.* the fibrillary assembly of collagen) resulting in mechanical properties that are quite far from physiology, or by being restricted to a fixed set of passive mechanical properties rather than mimicking dynamic changes (such as during development or fibrosis) or regional differences (such as within and outside MI areas). Closer interactions between materials scientists, synthetic biologists and cardiovascular experts are needed to drive the development of new experimental tools that more closely capture tissue-level dynamics, for example by enabling contact-free tuning of matrix stiffness (rapidly, reversibly, repeatedly, across a wide range or parameters, and with high spatial resolution). This will be a key area for research and development, as the principle role of spatial mechanical heterogeneities present in vivo has largely remained under-appreciated to date.

## Data Availability

The data that support the findings of this study are available from the corresponding author upon reasonable request.

## Abbreviations

3D: Three-dimensional
APD: Action potential duration
ARFI: Acoustic radiation force impulse
BK: ‘Big’ conductance potassium channel
CM: Cardiomyocyte
ECM: Extracellular matrix
EDPVR: End-diastolic pressure-volume relationship
FB: Fibroblast
HF: Heart failure
HFpEF: Heart failure with preserved ejection fraction
HFrEF: Heart failure with reduced ejection fraction
iPSC-CM: Induced pluripotent stem cell-derived cardiomyocyte
LV: Left ventricle
LTCC: L-type calcium channel
MB: Microbending
MD: Microfluidic deformability assay
MFB: Myofibroblast
MI: Myocardial infarction
MRE: Magnetic resonance elastography
MRI: Magnetic resonance imaging
NI: Nanoindentation
PVR: Pressure-volume relationship
SWE: Shear wave elastography
TT: Tensile testing

## Footnotes

1 There are no ‘muscle fibres’ in myocardium, unlike in skeletal muscles. The term is used as shorthand to refer to locally prevailing CM orientation.

2 CM are locally bundled into layers that are 3–5 cells thick. These are believed to be important to sustain in tissue shear, aiding myocardial wall thickening (Costa et al. 1999).
